# External quality assessment of dengue and chikungunya diagnostics in the Asia Pacific region, 2015

**DOI:** 10.5365/WPSAR.2016.7.1.002

**Published:** 2016-04-22

**Authors:** Li Ting Soh, Raynal C Squires, Li Kiang Tan, Kwoon Yong Pok, HuiTing Yang, Christina Liew, Aparna Singh Shah, John Aaskov, Sazaly Abubakar, Futoshi Hasabe, Lee Ching Ng, Frank Konings

**Affiliations:** a WHO Collaborating Centre for Reference and Research of Arbovirus and their Associated Vectors, Environmental Health Institute, National Environment Agency, Singapore.; b Emerging Disease Surveillance and Response, Division of Health Security and Emergencies, World Health Organization Regional Office for the Western Pacific, Manila, Philippines.; c Blood Safety and Laboratory Technology, Communicable Diseases Department, World Health Organization Regional Office for the South East-Asia, New Delhi, India.; d WHO Collaborating Centre for Arbovirus Reference and Research (Dengue/Severe Dengue), Tropical Infectious Diseases Research and Education Centre, Department of Medical Microbiology, Faculty of Medicine, University of Malaya, Kuala Lumpur, Malaysia.; e WHO Collaborating Centre for Reference and Research on Tropical and Emerging Virus Diseases, Nagasaki University, Nagasaki, Japan.; * Both authors contributed equally in the writing of this paper.

## Abstract

**Objective:**

To conduct an external quality assessment (EQA) of dengue and chikungunya diagnostics among national-level public health laboratories in the Asia Pacific region following the first round of EQA for dengue diagnostics in 2013.

**Methods:**

Twenty-four national-level public health laboratories performed routine diagnostic assays on a proficiency testing panel consisting of two modules. Module A contained serum samples spiked with cultured dengue virus (DENV) or chikungunya virus (CHIKV) for the detection of nucleic acid and DENV non-structural protein 1 (NS1) antigen. Module B contained human serum samples for the detection of anti-DENV antibodies.

**Results:**

Among 20 laboratories testing Module A, 17 (85%) correctly detected DENV RNA by reverse transcription polymerase chain reaction (RT–PCR), 18 (90%) correctly determined serotype and 19 (95%) correctly identified CHIKV by RT–PCR. Ten of 15 (66.7%) laboratories performing NS1 antigen assays obtained the correct results. In Module B, 18/23 (78.3%) and 20/20 (100%) of laboratories correctly detected anti-DENV IgM and IgG, respectively. Detection of acute/recent DENV infection by both molecular (RT–PCR) and serological methods (IgM) was available in 19/24 (79.2%) participating laboratories.

**Discussion:**

Accurate laboratory testing is a critical component of dengue and chikungunya surveillance and control. This second round of EQA reveals good proficiency in molecular and serological diagnostics of these diseases in the Asia Pacific region. Further comprehensive diagnostic testing, including testing for Zika virus, should comprise future iterations of the EQA.

Global dengue incidence has increased in recent decades, though the actual numbers of dengue cases are masked by underreporting. Bhatt et al. suggested that there are 390 million dengue virus (DENV) infections per year, of which 96 million manifest clinically. (*1*) Estimated to bear around 70% of the global burden, the Asia Pacific region (comprising the World Health Organization [WHO] South-East Asia and Western Pacific Regions) is an area of high dengue activity with multiple and large outbreaks occurring yearly. In the Western Pacific Region in 2014 alone, there were outbreaks involving 1513 dengue cases in Solomon Islands, (*2*) 45 171 cases in China and 108 698 cases in Malaysia. (*3*) Japan reported its first autochthonous outbreak in over 70 years (*4*, *5*) and DENV-serotype 3 was found to be circulating in the Pacific after an absence of 18 years. (*6*)

Chikungunya is an emerging threat to the Asia Pacific region. The disease is caused by the chikungunya virus (CHIKV), an alphavirus spread by some of the same mosquito vectors as DENV (*Aedes aegypti* and *Ae. albopictus*, among others). Clinical symptoms resemble dengue, and while chikungunya is a generally milder disease, debilitating sequelae such as persistent arthralgia have been reported in 36–64% of cases. (*7*) CHIKV has probably had an unappreciated circulation in the region due to its disease presentation and co-circulation with DENV. (*7*, *8*) That may also be the case for Zika virus (ZIKV), a flavivirus that was detected in Asia in the 1960s but has recently emerged in the Pacific and the Americas. (*9*) ZIKV has been linked to clusters of microcephaly and other neurological disorders that WHO declared on 1 February 2016 to constitute a public health emergency of international concern. (*10*)

Accurate laboratory diagnosis is a critical component of surveillance and response. The similarity of dengue and chikungunya symptoms makes differential diagnosis difficult without laboratory confirmation, especially in dengue-endemic areas. This impacts public health response as the *Ae. aegypti* and *Ae. albopictus* mosquito vectors require different control strategies, (*11*) and clinicians require specialized training to treat severe dengue cases. (*12*) Diagnostics for dengue and chikungunya are comparable. During the acute phase of infection, diagnosis focuses on detection of viral RNA (or DENV non-structural protein 1 [NS1]); immunoglobulin M (IgM) and/or high titre immunoglobulin G (IgG) antibodies are the diagnostic targets in the convalescent phase. (*7*, *13*) Despite the high initial cost, technical expertise and well equipped facilities required for RNA detection using reverse transcription polymerase chain reaction (RT–PCR), this platform permits simultaneous detection of multiple pathogens and generates serotype (DENV) and genotype data useful for tracking the movement of viruses and for risk assessment. (*14*) There are several commercial diagnostic tests for the detection of DENV and CHIKV by RT–PCR or for the detection of IgG and IgM antibodies against the viruses. However, while point-of-care tests for dengue diagnosis in non-clinical settings are well established, similar, reliable rapid diagnostic tests (RDTs) for chikungunya are not available. (*15*)

We recently reported the results of the first regional external quality assessment (EQA) for dengue in national-level public health laboratories in the WHO Western Pacific Region (*13*) that was initiated under the Asia Pacific Strategy for Emerging Diseases (APSED). (*16*) That 2013 study, based on the WHO existing influenza EQA programme (*17*) and using a small panel containing inactivated DENV and convalescent patient serum provided an initial overview of dengue diagnostic testing in the Region, revealing good proficiency in molecular and serological diagnostics. The current study reflects an expansion of the panel to comprise more samples for dengue diagnosis, the inclusion of CHIKV samples and a broader geographic coverage with the additional participation of national-level public health laboratories from the WHO South-East Asia Region.

## Methods

### Participating laboratories

Twenty-four national-level public health laboratories from 22 countries and areas in the WHO South-East Asia and Western Pacific Regions participated in this EQA (listed at end of article). The EQA panel was dispatched between February and May 2015.

### Preparation of EQA panel

The WHO Collaborating Centre for Reference and Research of Arbovirus and their Associated Vectors, located at the Environmental Health Institute of the National Environment Agency, Singapore, was selected as the EQA provider as it had the necessary technical expertise, access to samples and the required resources.

The 2015 EQA panel comprised two modules (A and B) containing 1 mL of serum spiked with inactivated DENV or CHIKV (Module A) and 0.2 mL of serum obtained from convalescent dengue patients (Module B) (**Table 1**). All patient samples were heat-treated at 56 °C for 1 hour and tested negative for human immunodeficiency virus, hepatitis B surface antigen and hepatitis C virus antibody.

**Table 1:** Characteristics of modules used in EQA of dengue and chikungunya diagnostics, WHO South-East Asia and Western Pacific Regions, 2015

Module	Sample ID	Contents	Serotype/strain and titre (GE/mL)*	Antibodies
Viral RNA/NS1 antigen (Module A)	A2015-V01	Inactivated DENV in serum	DENV-1 (1.1X10 (*6*))	–
	A2015-V02	Inactivated DENV in serum	DENV-1 (1.0X10 (*6*))	–
	A2015-V03	Inactivated DENV in serum	DENV-1 (1.7X10 (*5*))	–
	A2015-V04	Inactivated DENV in serum	DENV-1 (1.5X10 (*6*))	–
	A2015-V05	Inactivated DENV in serum	DENV-1 (1.4X10 (*6*))	–
	A2015-V06	Inactivated DENV in serum	DENV-1 (5.0X10 (*5*))	–
	A2015-V07	Serum alone	Negative control	–
	A2015-V08	Inactivated CHIKV in serum	ECSA (8.2X10 (*4*))	–
	A2015-V08	Inactivated CHIKV in serum	ECSA (9.9X10 (*4*))	–
	A2015-V10	Serum alone	Negative control	–
Antibody (Module B)	B2015-S01	Negative human serum	–	Negative control
	B2015-S02^†^	Convalescent serum	–	IgM, IgG
	B2015-S03^†^	Convalescent serum	–	IgM, IgG
	B2015-S04^†^	Convalescent serum	–	IgM, IgG
	B2015-S05^†^	Convalescent serum	–	IgM, IgG
	B2015-S06	Negative human serum	–	Negative control

* Virus titre is defined as average genomic copy number (genomic equivalents, GE) per mL, *n* = 5.

^†^ B2015-S02 and B2015-S03, and B2015-S04 and B2015-S05, were the same samples collected from two recently recovered dengue patients used to assess reproducibility of testing results.

CHIKV, chikungunya virus; DENV, dengue virus; ECSA, East Central and South African lineage; ID, identification; and NS1, non-structural protein 1.

For Module A, inactivated DENV and CHIKV isolates were prepared from mammalian cell culture (Vero and BHK Clone 21, respectively) supernatants of DENV-1 (SG(EHI)D1/19944Y13, Genotype III, GenBank: KP685234), DENV-3 (SG(EHI)D3/26592Y13, Genotype III, GenBank: KP685235) and CHIKV (SGEHICH06071Y13, GenBank: KP685237). Viral particles in cell supernatants were inactivated by heating at 60 °C for 1 hour and verified non-infective through three passages in an in-house, cell-based viral infectivity assay. Heat-treated samples were diluted in pathogen-free human serum (SeraCare Life Sciences, Milford, MA, USA) and the final viral loads (measured in genomic equivalents/millilitre [GE/mL]) of DENV and CHIKV were determined by an in-house real-time RT–PCR assay. (*18*, *19*) The presence of NS1 antigen in DENV samples was confirmed using commercial dengue NS1 assays (Panbio Dengue Early NS1 antigen capture ELISA [Alere Inc., Waltham, MA, USA] and Dengue NS1 Ag cassette [Standard Diagnostics Inc., Kyonggi-do, Republic of Korea]). Only samples containing DENV detectable by both NS1 and RT–PCR assays were included in the module. Two samples (A2015-V07 and A2015-V10) were included as negative controls (serum only) and were confirmed DENV- and CHIKV-negative by the real-time RT–PCR and commercial dengue NS1 assays mentioned above.

For Module B, the convalescent sera of two recently recovered dengue patients were split into two sets (B2015-S02 and B2015-S03; and B2015-S04 and B2015-S05). These sera contained neutralizing antibodies to DENV 1–4 (> 1:1000, as determined by an in-house cell-based plaque-reduction neutralization technique [PRNT]). (*20*) These samples also tested positive for the presence of dengue IgM and IgG antibodies using DENV commercial assays (Bioline Dengue Duo [Standard Diagnostics Inc.], Dengue Virus IgM Capture DxSelect [Focus Diagnostics, Cypress, CA, USA], and Panbio Dengue IgG Capture and IgG Indirect ELISA [Alere Inc.]). Samples B2015-S02 and B2015-S03 were designated as high IgM (> 72 Panbio units by IgM capture; positive is > 11) and high IgG (> 97 Panbio units by IgG capture; positive is > 22), while samples B2015-S04 and B2015-S05 were designated as low IgM (18 Panbio units by IgM capture; positive is > 11) and high IgG (> 87 Panbio units by IgG capture; positive is > 22). Two samples (B2015-S01 and B2015-S06) were included as negative controls (human sera only) and were confirmed negative for anti-DENV antibodies using the above-mentioned commercial and PRNT assays.

Before dispatch to participating laboratories, all EQA samples were tested by an independent International Organization for Standardization (ISO) 15189 and College of American Pathologists (CAP)-accredited laboratory, using DENV and CHIKV RT–PCR assays, (*21*, *22*) and the SD Bioline Dengue Duo kit [Standard Diagnostics Inc.].

Participating laboratories could subscribe to one or both modules. Individual samples were number-coded and frozen at −80 °C until dispatch. One laboratory requested and was provided with positive controls for the four DENV serotypes in its shipment to validate its dengue RT–PCR protocols. Similarly, laboratories were provided with a CHIKV-positive control as well as recommended references for conventional or real-time RT–PCR protocols and primer/probe sequences if they requested them.

### Data collection and analysis

Each participant was given a unique identifier to assure anonymous participation, an instruction form as well as results submission and feedback forms. Clinical notes accompanied Module B samples. Intentional sample labelling errors were included to assess the sample pre-processing measures of the participating laboratories. Laboratories were requested to examine the EQA samples by routine diagnostic methods; report any clerical errors identified; and submit background technical information on methods, kits, protocols and reagents used.

In Module A, two points each were awarded for the correct detection of DENV either by RT–PCR or NS1 assays, correct serotyping of DENV and correct detection of CHIKV by RT–PCR. In Module B, two points each were awarded for the correct detection of dengue IgM and IgG antibodies. All (including complementary) assays performed were scored; no penalty was applied for assays not done. Equivocal results submitted for true positive samples were awarded one point. (*23*) Using in-date reagents or validating expired reagents earned up to four additional points for each module. Identification of intentional clerical errors scored an additional point for each module. The final score was the proportion of points earned out of the possible awardable points.

## Results

### Overall laboratory proficiency

**Fig. 1:**
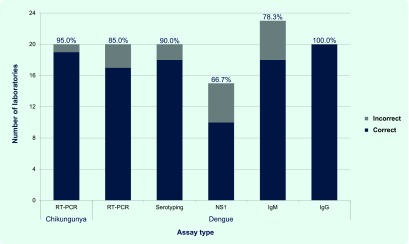
Proportion of participating laboratories by test conducted and results, EQA of dengue and chikungunya diagnostics, WHO South-East Asia and Western Pacific Regions, 2015

*Note:* Percentage of laboratories performing each test correctly is displayed above columns.

NS1, non-structural protein 1; and RT–PCR, reverse transcription polymerase chain reaction.

Twenty-four laboratories participated in this 2015 EQA, with 20 and 23 laboratories testing Modules A and B, respectively. Nineteen laboratories tested both. Overall results are presented in **Fig. 1**. The majority of participants detected DENV (17/20, 85%), DENV serotype (18/20, 90%) and CHIKV (19/20, 95%) by RT–PCR correctly. Accuracy was moderate (10/15, 66.7%) for NS1 testing. The most commonly performed EQA component was anti-DENV IgM detection with 18/23 (78.3%) laboratories reporting correct results. Twenty laboratories detected anti-DENV IgG in samples with 100% accuracy. Seven laboratories performed complementary assays for a single sample type (this approach aids in eliminating false positives or negatives in routine diagnostics) and reported correct results for at least one of the assays used (**Table 2**). Eighteen of 24 laboratories (75%) failed to identify the intentional clerical errors on sample labels.

**Table 2:** Performance summary of participating laboratories, EQA of dengue and chikungunya diagnostics, WHO South-East Asia and Western Pacific Regions, 2015

Laboratory identifier	1301	1302	1303	1304	1306	1307	1308	1309	1310	1311	1312	1313	1314	1315	1316	1317	1318	1319	1420	1421	1422	1423	1424	1425
*Nucleic acid detection (RT–PCR)*																								
DENV																								
Real-time	●	●	●			●		●	●	●	○			●	●	●		●	●					
Conventional	●			●	○							●	●				○						●	●
DENV serotyping																								
Real-time	●	●	●			○		●	●	●	●			●	●	●		●	●					
Conventional	●			●	●							●	●				●						○	●
CHIKV																								
Real-time	●	●	●			○		●	●		●	●		●	●	●		●	●				●	
Conventional				●	●					●			●			●	●							●
*DENV NS1 antigen detection*																								
ELISA	○			●		●		●						●	○	○	●	○	○				○	
RDT	●	●		●	●					●			○	○					●					
*Anti-DENV IgM detection*																								
ELISA	●	●	●	●		●	●	●	●	○		●	○	●	●	○	○	●	○	●	●	●	●	●
RDT	●	●			●											●						●		
*Anti-DENV IgG detection*																								
ELISA	●		●	●		●			●	●		●	●	●			●	●	●	●			●	●
RDT	●	●			●											●						●		
HI	●														●	●								
Module shipment (days)	2	1	3	2	1	8	2	3	3	2	4	1	2	3	1	1	2	2	2	6	1	1	8	2
Testing turnaround (days)	41	27	36	72	54	29	41	7	25	27	28	12	30	32	21	27	49	30	27	28	13	24	50	44

*Note*: Filled circles indicate correct results for all samples; open circles indicate incorrect results for at least one sample.

CHIKV, chikungunya virus; DENV, dengue virus; ELISA, enzyme-linked immunosorbent assay; HI, haemagglutination inhibition assay; NS1: non-structural protein 1; RDT, rapid diagnostic test; and RT–PCR, reverse transcription polymerase chain reaction.

### Module A: Viral RNA and NS1 antigen

Of the 20 laboratories performing RT–PCR in Module A, 15 (75%) used real-time RT–PCR technology for nucleic acid detection at some point during their testing and 10 laboratories (50%) used it exclusively (**Table 2**). Few laboratories demonstrated errors in detection of DENV, DENV serotype or CHIKV by RT–PCR. Of the three laboratories with errors in DENV detection, two using conventional RT–PCR reported the DENV-positive samples (A2015-V01, V02 and V03) as negative and one laboratory using real-time RT–PCR methodology reported the DENV-positive samples (A2015-V01, V02 and V06) as negative. Of the two laboratories exhibiting serotyping errors, one reported both DENV-1 and DENV-4 in a DENV-1-only sample (A2015-V03) and another reported the presence of DENV-4 in two DENV-3 samples (A2015-V04 and A2015-V05). One laboratory detected CHIKV in a serum-only sample (A2015-V10). DENV genome regions targeted for virus detection and serotyping varied with *capsid* and *non-structural protein 5* being the most common. CHIKV detection targets included the *envelope 1*, and the *non-structural protein 1* and *4* genes.

Fifteen laboratories performed NS1 antigen detection assays using the ELISA methodology alone (7/15), both ELISA and commercial RDT (4/15) or RDT alone (4/15). Five laboratories performing ELISA on DENV-positive sample A2015-V03 using the Platelia Dengue NS1 Ag kit (Bio-Rad Laboratories, Inc., Hercules, CA, USA) demonstrated errors; four reported equivocal results and one reported a false-negative result.

### Module B: Serology

Anti-DENV IgM assays were performed by all 23 laboratories testing Module B using the ELISA methodology alone (18/23), both ELISA and RDT (4/23) or RDT alone (1/23) (**Table 2**). Antibody capture ELISAs from Panbio (Alere Inc.) and SD (Standard Diagnostics Inc.) were the most commonly employed assays for IgM detection. Of the 22 laboratories performing anti-DENV IgM ELISAs, 17 (77.3%) obtained correct results, while five (22.7%) reported equivocal or false-negative results for at least one of two IgM-positive samples (B2015-S04 and B2015-S05). No errors were reported among RDT users.

Twenty laboratories tested for both anti-DENV IgG and IgM in Module B; only three tested for IgM alone. Anti-DENV IgG was correctly detected by all methods used. Fourteen (70%) laboratories employed ELISA assays alone for IgG detection, while the remainder used a commercial RDT kit, a haemagglutination inhibition assay (HI), or both.

### Comparison with the 2013 EQA

**Fig. 2:**
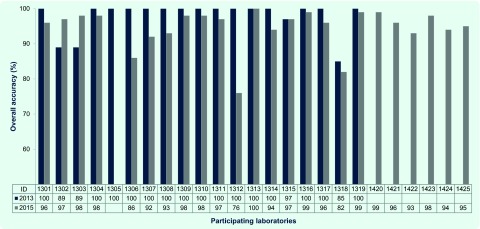
Overall accuracy (final score) of participating laboratories in the 2013 and 2015 WHO EQAs of dengue and chikungunya diagnostics

Of the 18 laboratories that participated in the 2013 (*13*) and 2015 EQAs, four (22%) were able to maintain or improve their overall score (expressed as percentage) in this EQA, while the final score in the remaining 14 laboratories fell by a median of 3.5% (**Fig. 2**). Scores for the majority (12/14) of these laboratories fell by ≤ 8%. In contrast, scores for two laboratories fell by 14% and 24%, and another, repeating the same serology detection error made in 2013, scored consistently low (≤ 85%) in both years.

ID, laboratory identifier.

### Logistics

The average turnaround time for result submission was 32 days. The 20 laboratories requesting CHIKV samples were offered an additional 30 days to optimize their CHIKV RT–PCR protocols. Of the 13 laboratories accepting the extension, five used it, including two laboratories with a 14-day national holiday within their testing periods. One laboratory requested a 24-day extension due to shortage of reagents.

There were no major logistical challenges associated with shipment of test samples to participating laboratories. All samples arrived frozen at the time of receipt. Nearly all participating laboratories received test samples within four days; three laboratories received them in up to eight days due to extended customs clearance times.

## Discussion

This study reports on the second iteration of the WHO EQA for dengue diagnostics for national-level public health laboratories in the Western Pacific Region that has been expanded to include chikungunya diagnostics as well as national-level laboratories from the South-East Asia Region.

The appropriate dengue diagnostic tools must be used at the correct time for the correct diagnosis of dengue. While 19/24 (79.2%) laboratories employed assays for both acute (RT–PCR) and recent (anti-DENV IgM) DENV infection, four performed antibody testing for dengue but lacked assays for early detection (pre-antibody immunological response) of dengue such as RT–PCR or NS1 kits and one could perform RT–PCR but had no serology capacity. With an incomplete set of diagnostics, these laboratories may be unable to diagnose a proportion of DENV infections and should consider quickly strengthening their capacity through the use of commercial ELISA assays for the detection of NS1 antigen or anti-DENV IgM antibodies.

Accuracy was high (≥ 85%) for DENV and CHIKV detection and DENV serotyping by RT–PCR. The few errors in DENV detection (false-negatives) appeared to be clustered in samples A2015-V01 and V02, which were identical, high-titre DENV-1 samples. Most of the inaccuracies in Module A were in NS1 testing. Specifically, 87.5% of NS1 testing errors were derived from a single DENV-positive sample, A2015-V03, being reported as negative or equivocal (7/8 laboratories), particularly when using the Platelia Dengue NS1 Ag kit. This suggests that the NS1 levels in the sample may have been at the threshold of detection for the kit, making complementary assays for virus detection, such as RT–PCR, highly relevant.

This EQA served as a platform for building capacity for RT–PCR detection of CHIKV. Of the 20 laboratories performing RT–PCR, 16 (80%) requested receipt of a CHIKV-positive control and 12 (60%) requested real-time or conventional RT–PCR protocols to develop and validate their capacity for CHIKV diagnosis.

Laboratories also performed anti-DENV IgM detection with good accuracy (78.3%). Errors in this component of the panel were the result of equivocal or false-negative ELISA results for samples B2015-S04 and/or B2015-S05. These were duplicate low titre IgM samples from a convalescent volunteer, suggesting that some laboratories should review the cut-off values of their anti-DENV IgM assays. All assays for detection of anti-DENV IgG were performed without error. Similar to the 2013 EQA, laboratories appeared to use either high titre IgG ELISAs suitable for detecting acute/recent infections or low titre IgG ELISAs for the detection of a prior dengue infection (such as in seroprevalence studies). Laboratories should be aware of the constraints of their IgG assays, but as Module B contained only high titre IgG samples, the recognition of these operational constraints could not be tested.

For laboratories participating in the 2013 and 2015 EQAs, a minimal decrease in final score on this round was anticipated, and was likely due to increased panel complexity as previously suggested. (*24*) But while improving on the first iteration of the EQA in several ways, this second round also had limitations. Module A contained 10 test samples as opposed to three in 2013 and included both DENV and CHIKV; however, the module comprised just two DENV serotypes and one CHIKV strain. This round of the EQA also prioritized concomitant sample testing by both DENV NS1 and RT–PCR assays as the NS1 RDT, while less sensitive than RT–PCR, is a key diagnostic and epidemiological tool for detection of acute DENV infection in clinical settings. However, this meant that DENV titres below 10^5^ GE/mL could not be introduced to gauge the sensitivity of RT–PCR assays. This was not so for the CHIKV samples that were titred at 10^4^ GE/mL and detected with high accuracy compared to similar samples in another EQA. (*25*) Future iterations of our study could place more emphasis on the sensitivity of DENV molecular testing by using lower titres considered suitable for surveillance and diagnostic purposes. (*26*)

Module B comprised high and low titre anti-DENV IgM samples but no low titre anti-DENV IgG samples that could be used to assess the sensitivity of IgG assays. The module also did not include any samples for serological detection of anti-CHIKV antibodies, though a subsequent study suggests this capacity is widely in place in the Western Pacific Region. (*27*) Additionally, that most laboratories failed to report the clerical errors included in the EQA suggested that instructions in future rounds should place greater emphasis on the importance of this quality control measure. Together, these limitations can be used as opportunities in subsequent rounds of this EQA, along with the introduction of testing for ZIKV. Given ZIKV’s link to serious neurological disorders and its co-circulation with DENV and CHIKV that cause similar symptoms, differential testing for these pathogens is crucial. (*27*)

This second round of EQA demonstrated that good proficiency in dengue and chikungunya diagnostics is in place in the Asia Pacific region. Laboratories demonstrating lower proficiency may be able to benefit from technical assistance available through this EQA. Future iterations of the EQA, featuring increased complexity and the inclusion of other priority pathogens, including ZIKV, will continue to contribute to strengthening regional laboratories’ diagnostic capacities for emerging diseases in line with APSED.
